# Polymorphisms in the *AKT1* and *AKT2* genes and oesophageal squamous cell carcinoma risk in an Eastern Chinese population

**DOI:** 10.1111/jcmm.12750

**Published:** 2016-02-01

**Authors:** Jinhong Zhu, Mengyun Wang, Jing He, Meiling Zhu, Jiu‐Cun Wang, Li Jin, Xiao‐Feng Wang, Ya‐Jun Yang, Jia‐Qing Xiang, Qingyi Wei

**Affiliations:** ^1^ Cancer Institute Fudan University Shanghai Cancer Center Shanghai China; ^2^ Department of Oncology Shanghai Medical College Fudan University Shanghai China; ^3^ Molecular Epidemiology Laboratory and Department of Laboratory Medicine Harbin Medical University Cancer Hospital Harbin Heilongjiang China; ^4^ Department of Pediatric Surgery Guangzhou Women and Children's Medical Center Guangzhou Medical University Guangzhou China; ^5^ Department of Oncology Xinhua Hospital Affiliated to Shanghai Jiaotong University, School of Medicine Shanghai China; ^6^ Ministry of Education Key Laboratory of Contemporary Anthropology State Key Laboratory of Genetic Engineering School of Life Sciences Fudan University Shanghai China; ^7^ Fudan‐Taizhou Institute of Health Sciences Taizhou Jiangsu China; ^8^ Department of Thoracic Surgery Fudan University Shanghai Cancer Center Fudan University Shanghai China; ^9^ Duke Cancer Institute Duke University Medical Center Durham NC USA

**Keywords:** *AKT1*, *AKT2*, oesophageal squamous cell carcinoma, risk, polymorphism

## Abstract

Ethnic Han Chinese are at high risk of developing oesophageal squamous cell carcinoma (ESCC). Aberrant activation of the AKT signalling pathway is involved in many cancers, including ESCC. Some single nucleotide polymorphisms (SNPs) in genes involved in this pathway may contribute to ESCC susceptibility. We selected five potentially functional SNPs in *AKT1* (rs2494750, rs2494752 and rs10138277) and *AKT2* (rs7254617 and rs2304186) genes and investigated their associations with ESCC risk in 1117 ESCC cases and 1096 controls in an Eastern Chinese population. None of individual SNPs exhibited an association with ESCC risk. However, the combined analysis of three *AKT1* SNPs suggested that individuals carrying one of *AKT1* variant genotypes had a decreased ESCC risk [adjusted odds ratio (OR) = 0.60, 95% CI = 0.42–0.87]. Further stratified analysis found that *AKT1* rs2294750 SNP was associated with significantly decreased ESCC risk among women (adjusted OR = 0.63, 95% CI = 0.43–0.94) and non‐drinkers (OR = 0.79, 95% CI = 0.64–0.99). Similar protective effects on women (adjusted OR = 0.56, 95% CI = 0.37–0.83) and non‐drinker (adjusted OR = 0.75, 95% CI = 0.60–0.94) were also observed for the combined genotypes of *AKT1* SNPs. Consistently, logistic regression analysis indicated significant gene–gene interactions among three *AKT1* SNPs (*P* < 0.015). A three‐*AKT1* SNP haplotype (C‐A‐C) showed a significant association with a decreased ESCC risk (adjusted OR = 0.70, 95% CI = 0.52–0.94). Multifactor dimensionality reduction analysis confirmed a high‐order gene–environment interaction in ESCC risk. Overall, we found that three *AKT1* SNPs might confer protection against ESCC risk; nevertheless, these effects may be dependent on other risk factors. Our results provided evidence of important gene–environment interplay in ESCC carcinogenesis.

## Introduction

Oesophageal cancer, consisting of squamous cell carcinoma (ESCC) and adenocarcinoma, is the 8th most frequently diagnosed cancer worldwide 1, 2, 3. Oesophageal squamous cell carcinoma constitutes the majority of the cases (90%) in China with a 5‐year survival of less than 20% 2, 3. Therefore, it is urgent to develop more effective prevention strategies for this malignant disease by a better understanding of the aetiology.

The proven‐environmental (*e.g*. lifestyle) risk facts for ESCC are poor nutritional status, low intake of fruits and vegetables, tobacco smoking, alcohol use and drinking hot beverages. Moreover, genetic factors are also implicated in ESCC carcinogenesis. Molecular epidemiological studies have demonstrated that some single nucleotide polymorphisms (SNPs) account, in part, for the variation in cancer susceptibility in the general population 4, 5, 6, including SNPs in inflammatory response, one carbon metabolism, metabolism of chemical carcinogens and DNA repair pathways as well as some other oncogenes and tumour‐suppressor genes 7, 8.

Over‐activation of the PI3K–AKT pathway has been implicated in the development of various human cancers, including cancers of the endometrium, stomach, lung and oesophagus 9, 10, 11, 12, 13, 14, 15, 16, 17. Once activated, phosphatidylinositols (PtdIns) 3‐kinases (PI3Ks), phosphorylates PtdIns (4,5) P2 (PIP2) to form PtdIns (3,4,5)P3 (PIP3), a second messenger. The PIP3 thereafter recruits AKT to the plasma membrane to facilitate AKT phosphorylation at Thr308 and Ser473. Activated AKT may trigger a series of biological effects on cells, involving survival, adhesion, motility, proliferation and growth, to stimulate malignant transformation of cells and tumour progression 18. AKT (alias: protein kinase B), the human homologue of the viral oncogene *v‐akt*, is one of well‐characterized key components of the PI3K–AKT signalling pathway. There are three known AKT isoforms (AKT1, AKT2 and AKT3) in mammals, which are encoded by distinct genes (*AKT1/PKB*α, *AKT2/PKB*β and *AKT3/PKB*γ, respectively). Given the important role of AKT in carcinogenesis, it is reasonably speculated that potentially functional SNPs in *AKT* genes may alter its expression and/or protein function, thereby modifying cancer susceptibility.

Many studies 9, 19, 20, 21, 22 have investigated the effects of SNPs in *AKT* genes on the risk of cancers in Chinese and shown promising results. However, the contribution of *AKT* polymorphisms to ESCC risk has not been reported. Therefore, we conducted this case–control study to explore the role of SNPs in *AKT* genes in the aetiology of ESCC in an Eastern Chinese population.

## Materials and methods

### Study population

This case–control study included 1117 cases and 1096 healthy non‐cancer controls. All enrolled cases were newly diagnosed ESCC patients between March 2009 and September 2011, with histopathological confirmation at Fudan University Shanghai Cancer Center. They were all genetically unrelated Han Chinese, residing in Eastern China. Exclusion criteria were as follows: (*i*) the primary tumour was not oesophageal in origin, (*ii*) patients with other cancers and (*iii*) cancers without a definite primary site. Cancer‐free controls (without other diseases) were from a large prospective cohort recruited for the Taizhou longitudinal study at the same time period in the Eastern China 23, and frequency matched to cases on age (±5 years) and sex. While interviewed, all participants were obligated to complete a structured questionnaire including demographic data and environmental exposure history, such as age, sex, ethnicity, body mass index (BMI, calculated by weight in kilograms/height^2^ in metres), tobacco use and alcohol intake before treatment. A BMI value of 25 was used as a cut‐off point to split participants into two groups with BMI <25 and ≥25, as the World Health Organization suggested BMI ≥25 as a cut‐off for classification of overweight 24. Only study participants who signed a written consent form (about 90%) were included in the final analysis. The research protocol of the study was approved by the institutional review board of the Fudan University Shanghai Cancer Center.

### SNP selection and genotyping

We first retrieved available SNPs in target genes from the National Center for Biotechnology Information dbSNP database (http://www.ncbi.nlm.nih.gov/projects/SNP) and then selected common, potentially functional SNPs in accordance with these criteria: (*i*) positioned in exons, the 5′ near gene, 5′ untranslated regions (UTR), 3′ UTR, 3′ near gene or splice sites; (*ii*) the minor allele frequency (MAF) should be equal or larger than 5% in Chinese Han population; (*iii*) SNPinfo software (http://snpinfo.niehs.nih.gov/snpfunc.htm)‐identified potentially functional SNPs; and (*iv*) not studied in the published ESCC genome‐wide association studies. Moreover, some SNP reported by others was also selected 25. Haploview software was used to check the linkage disequilibrium (LD) to ensure that selected SNPs were in low LD (γ^2^ < 0.8) with one another. Ultimately, five SNPs (*AKT1*: rs2494750, rs2494752 and rs10138277; *AKT2*: rs7254617 and rs2304186) were included in the study. No SNPs in the *AKT3* gene met the defined criteria and thus were not included.

Qiagen Blood DNA Mini Kit (Qiagen Inc., Valencia, CA, USA) was used to acquire genomic DNA from blood specimens, and TaqMan assay was performed to genotype DNA samples as indicated previously 26. Concisely, allele‐specific probes for SNP genotyping were purchased from Applied Biosystems (Foster City, CA, USA). For each of selected SNPs, the probes for the variant and wild‐type alleles were labelled with either of the fluorescent dyes VIC and FAM, respectively. The ABI 7900 HT Sequence Detection System (Applied Biosystems) allowed the use of a post‐amplification allelic discrimination run on the machine to identify genotype according to the relative fluorescence intensity of VIC and FAM. PCR reactions in 384‐well plates was run on the machine, with a total reaction volume of 5 μl for each sample. Individuals involved in genotyping were blind to participants' status.

### *AKT1* expression analysis based on *AKT1* variant genotypes

We further interrogated the impact of the significant polymorphisms on the gene expression by using online databases for 270 individuals from four worldwide populations [CEU: 90 Utah residents with ancestry from northern and western Europe; CHB: 45 unrelated Han Chinese in Beijing; JPT: 45 unrelated Japanese in Tokyo; YRI: 90 Yoruba in Ibadan, Nigeria] 27. We first obtained genotype information from the international HapMap phase (II+III) release #28 data set, containing genotype data of 3.96 million polymorphisms for 270 individuals (http://www.hapmap.org). mRNA expression information was acquired from the same 270 individuals (http://app3.titan.uio.no/biotools/help.php?app=snpexp) 28, which were derived from GENe Expression VARiation (http://www.sanger.ac.uk/resources/software/genevar/) 29. Finally, we matched *AKT1* polymorphism genotypes and *AKT1* mRNA expression levels for each individual to evaluate the correlation between Hapmap genotypes and the gene expression levels.

### Statistical methods

The chi‐squared test was used to evaluate whether there was any difference in the frequency distributions of certain demographic variables, risk factors and genotypes of the studied SNPs between the cases and controls. A goodness‐of‐fit chi‐squared test was used to detect possible deviation from Hardy–Weinberg equilibrium (HWE) in controls. The crude and adjusted odds ratios (ORs) and 95% confidence intervals (CIs) for the association of ESCC risk with SNPs of interest were determined by univariate and multivariate logistic regression analyses controlling for co‐variates (*e.g*. age, sex, smoking, drinking and BMI). The stratification analyses were also performed to identify the associations by age, sex, BMI, and smoking and drinking status. Moreover, a combination of rs2494750, rs2494752 and rs10138277 genotypes in the *AKT1* gene was considered as a haplotype. Unphased genotype data were used to determined haplotype frequencies and individual haplotypes. Logistic regression analysis was performed to calculate ORs and 95% CIs for the association of haplotypes with ESCC risk. All tests were two‐sided with a significance level of *P* < 0.05. All statistical analyses were performed with SAS software (version 9.1; SAS Institute, Cary, NC, USA). Furthermore, the high‐order gene–gene or gene–environment interactions were established in the association with cancer risk using the multifactor dimensionality reduction (MDR) software (V2.0 beta 8.2), as described elsewhere 30. A model with the minimum average prediction error and the maximum cross‐validation consistency (CVC) was considered the best candidate interaction model.

Finally, we performed mini meta‐analyses to evaluate the association of *AKT1* rs2494750 and *AKT2* rs7254617 SNPs with ESCC risk. Briefly, relevant studies were searched with defined search terms from the common public database (MEDLINE and EMBASE) and screened with inclusion and exclusion criteria in accordance with previous procedure 31, 32, 33. Chi‐square‐based *Q*‐test was performed to test heterogeneity assumption. The fixed‐effects model (the Mantel–Haenszel method) was used to calculate the pooled OR estimates. If the study had high heterogeneity, the random‐effects model (the DerSimonian and Laird method) would be chosen as an alternative. The funnel plot and Egger's linear regression test were used while detecting potential publication bias. Sensitivity analysis was performed to assess the effect of single studies on pooled risk estimates. We were not able to perform meta‐analysis for the remaining SNPs, because of very few publications having investigated the association of these SNPs and cancer risk. All the statistical tests were performed with STATA (version 11.0; Stata Corporation, College Station, TX, USA). Two‐sided *P*‐values were applied, and a *P* < 0.05 was used as the significance level.

## Results

### Characteristics of ESCC patients and controls

In this study, cases and controls were well matched by age (*P* = 0.338) and sex (*P* = 0.072; Table 1). Distributions of smokers and drinkers were found to be significantly different between cases and controls. As expected, the percentages of smokers and drinkers in cases were higher than in controls (smokers: 61.2% *versus* 54.2%, *P* < 0.0009; drinkers: 44.3% *versus* 32.9%, *P* < 0.0001). Moreover, mean BMI was significantly smaller in cases than in controls (mean BMI ± SD: 23.46 ± 7.36 *versus* 26.88 ± 7.52, *P* < 0.0001). Along with univariate analyses, multivariate logistic regression analyses adjusted for these variables were subsequently performed to control for potential confounding effect.

**Table 1:** Frequency distributions of selected characteristics of ESCC cases and cancer‐free controls in an Eastern Chinese population

Variables	Cases, no. (%)	Controls, no. (%)	*P* a
All participants	1117 (100.0)	1096 (100.0)	
Age, year			
Mean^b^	60.4 ± 8.3	60.8 ± 10.6	0.338
Age group			
≤50	138 (12.4)	152 (13.9)	
51–60	419 (37.5)	391 (35.7)	
61–70	424 (38.0)	384 (35.0)	
>70	135 (12.2)	169 (15.4)	
Sex			
Males	907 (80.8)	851 (77.7)	0.072
Females	215 (19.3)	245 (22.4)	
Drinking status			
Ever	495 (44.3)	360 (32.9)	<0.0001
Never	622 (55.7)	736 (67.1)	
Smoking status			
Ever	684 (61.2)	594 (54.2)	<0.0009
Never	433 (38.8)	502 (45.8)	
Pack‐years			
0	429 (38.4)	502 (45.8)	<0.0001
≤16 (mean)	148 (13.3)	239 (21.8)	
>16 (mean)	540 (48.3)	355 (32.4)	
Body mass index			
<25.0	714 (63.9)	487 (44.4)	<0.0001
≥25.0	403 (36.1)	609 (55.6)	

a Two‐sided chi‐squared test for distributions between cases and controls.

b Data were presented as mean ± S.D.

### Association between *AKT1*/*AKT2* SNPs and ESCC susceptibility

First, the genotype distributions of the five SNPs in controls were consistent with those expected from the HWE. Second, the MAFs of the genotyped SNPs in controls were comparable to those identified in the CHB data from HapMap or reported in Asians 25: 0.315 *versus* 0.267 (rs2494750), 0.266 *versus* 0.220 (rs2494752), 0.104 *versus* 0.083 (rs10138277), 0.135 *versus* 0.149 (7254617) and 0.447 *versus* 0.54 (rs2304186). We calculated ORs using logistic regression analyses after adjustment for age, sex, drinking status, smoking status and BMI (Table 2). In the single‐locus analysis, comparison of genotype frequency distributions revealed no significant difference between ESCC cases and controls, indicating that none of these SNPs was independently associated with ESCC risk in this study population.

**Table 2:** Logistic regression analysis of associations between the genotypes of *AKT1&AKT2,* and ESCC cancer risk

Variants	Genotypes	Cases (*N* = 1117)	Controls (*N* = 1096)	*P* a	Crude OR (95% CI)	*P*	Adjusted OR (95% CI)	*P* b
*AKT1* rs2494750	GG	555 (49.7)	521 (47.5)	0.595‡	1.00		1.00	0.302
	CG	448 (40.1)	460 (42.0)		0.91 (0.77–1.09)	0.320	0.90 (0.75–1.08)	0.245
	CC	114 (10.2)	115 (10.5)		0.93 (0.70–1.24)	0.621	0.89 (0.66–1.10)	0.431
	CG/CC	562 (50.3)	575 (52.5)		0.92 (0.78–1.08)	0.312	0.90 (0.75–1.06)	0.210
	CG/GG	1003 (89.8)	981 (89.5)		1.00		1.00	
	CC	114 (10.2)	115 (10.5)		0.97 (0.74–1.28)	0.825	0.93 (0.70–1.24)	0.634
*AKT1* rs2494752	AA	611 (54.7)	597 (54.5)	0.978‡	1.00		1.00	0.610
	AG	423 (37.9)	415 (37.9)		1.00 (0.84–1.19)	0.964	0.99 (0.83–1.20)	0.913
	GG	83 (7.4)	84 (7.6)		0.96 (0.70–1.33)	0.831	0.90 (0.64–1.25)	0.512
	AG/GG	506 (45.3)	499 (45.5)		0.99 (0.84–1.17)	0.914	0.97 (0.82–1.16)	0.760
	AG/AA	1034 (92.6)	1012 (92.4)		1.00		1.00	
	GG	83 (7.4)	84 (7.6)		0.97 (0.71–1.33)	0.835	0.90 (0.65–1.25)	0.518
*AKT1* rs10138277	CC	898 (80.4)	878 (80.1)	0.986‡	1.00		1.00	0.670
	CT	209 (18.7)	208 (19.0)		0.98 (0.79–1.22)	0.871	0.95 (0.76–1.18)	0.640
	TT	10 (0.9)	10 (0.9)		0.98 (0.41–2.36)	0.960	0.99 (0.40–2.45)	0.986
	CT/TT	219 (19.6)	218 (19.9)		0.98 (0.80–1.21)	0.867	0.95 (0.77–1.18)	0.647
	CT/CC	1107 (99.1)	1086 (99.1)		1.00		1.00	
	TT	10 (0.9)	10 (0.9)		0.98 (0.41–2.37)	0.966	1.00 (0.40–2.49)	0.997
*AKT2* rs7254617	GG	831 (74.4)	825 (75.2)	0.645‡	1.00		1.00	0.946
	AG	265 (23.7)	246 (22.5)		1.07 (0.88–1.30)	0.507	1.06 (0.86–1.30)	0.567
	AA	21 (1.9)	25 (2.3)		0.83 (0.46–1.50)	0.546	0.78 (0.42–1.45)	0.431
	AG/AA	286 (25.6)	271 (24.8)		1.05 (0.87–1.27)	0.634	1.04 (0.85–1.27)	0.728
	AG/GG	1096 (98.1)	1071 (97.7)		1.00		1.00	
	AA	21 (1.9)	25 (2.3)		0.82 (0.46–1.48)	0.510	0.77 (0.41–1.43)	0.403
*AKT2* rs2304186	GG	348 (31.2)	339 (30.9)	0.993‡	1.00		1.00	0.766
	GT	543 (48.6)	535 (48.8)		0.99 (0.82–1.20)	0.907	1.03 (0.84–1.25)	0.787
	TT	226 (20.2)	222 (20.3)		0.99 (0.79–1.26)	0.945	1.04 (0.81–1.36)	0.781
	GT/TT	769 (69.9)	757 (69.1)		0.99 (0.83–1.20)	0.909	1.03 (0.85–1.24)	0.756
	GT/GG	891 (79.8)	874 (79.7)		1.00		1.00	
	TT	226 (20.2)	222 (20.3)		1.00 (0.81–1.23)	0.989	1.02 (0.82–1.26)	0.867
Combined effect of *AKT1* variant genotypes	0	553 (49.51)	510 (46.53)		1.00		1.00	
	1	58 (5.19)	86 (7.85)		**0.62 (0.44–0.89)**	**0.009**	**0.60 (0.42–0.87)**	**0.007**
	2	289 (25.9)	294 (26.8)		0.91 (0.74–1.11)	0.342	0.90 (0.73–1.11)	0.324
	3	217 (19.43)	206 (18.80)		0.97 (0.78–1.22)	0.802	0.93 (0.74–1.18)	0.555
					*P*_trend_ = 0.607		*P*_trend_ = 0.430	
	0	553 (49.51)	510 (46.53)		1.00		1.00	
	≥1	564 (50.49)	586 (53.47)		0.89 (0.75–1.05)	0.162	0.87 (0.73–1.03)	0.108

a Chi‐squared test for genotype distributions between cases and controls.

b Adjusted for age, sex, BMI, smoking and drinking status in logistic regress models.

The results were in bold, if the 95% CI excluded 1 or *P* < 0.05.

CI, confidence interval; OR, odds ratio.

Next, we explored whether combined analysis of multiple genetic variants facilitated the identification of high‐risk individuals. We combined variant genotypes of the five SNPs (variant heterozygotes and homozygotes) under investigation to scrutinize whether these SNPs would collaboratively contribute to ESCC risk. Once again, participants carrying one to five variant genotypes have ESCC risk as high as those carrying wild‐type genotypes. Furthermore, all participants were split into two groups based on the presence or absence of variant genotypes, with one group having only the wild‐type genotype as reference and the other having at least one variant genotype. Likewise, we found carriers of one or more variant genotypes did not show altered risk (OR = 0.94, 95% CI = 0.68–1.28, *P* = 0.683) for ESCC, when compared with non‐carriers. However, the combined analysis with only three *AKT1* SNPs found that having one *AKT1* variant genotype was associated with a protective effect (adjusted OR = 0.60, 95% CI = 0.42–0.87, *P* = 0.007, statistical power = 0.353) for developing ESCC, which is likely because of a chance.

### Stratification analysis

We thereafter explored the gene–environment interaction by determining the potential association of ESCC risk with the SNPs in the stratified analyses by age, sex, smoking status, drinking status and BMI. Among all the tested SNPs, we found that *AKT1* rs2294750 might exert a protective effect on ESCC risk; in particular, this effect was significant for women (adjusted OR = 0.63, 95% CI = 0.43–0.94, *P* = 0.024, statistical power = 0.925) and non‐drinkers (OR = 0.79, 95% CI = 0.64–0.99, *P* = 0.042, statistical power = 0.995) under the dominant model (Table 3A). Moreover, the stratification analyses did not identify any other significant association (Table 3A and B).

**Table 3:** Stratification analysis for the associations between *AKT1* variant genotypes and ESCC risk

Variables	rs2494750							Combined variant genotypes						
	(cases/controls)		Crude OR(95% CI)	*P*	Adjusted OR^a^ (95% CI)	*P* a	*P*_*hom*_	(cases/controls)		Crude OR (95% CI)	*P*	Adjusted OR^a^ (95% CI)	*P* a	*P*_*hom*_
	GG	GC/CC						0	≥1					
Age														
≤60	286/265	271/278	0.90 (0.71–1.14)	0.399	0.88 (0.69–1.13)	0.330	0.852	286/260	271/283	0.87 (0.69–1.10)	0.251	0.86 (0.67–1.10)	0.228	0.818
>60	269/256	291/297	0.93 (0.74–1.18)	0.560	0.92 (0.72–1.17)	0.500		267/250	293/303	0.91 (0.72–1.15)	0.409	0.89 (0.70–1.13)	0.344	
Sex														
Females	118/114	97/131	0.72 (0.50–1.03)	0.074	**0.63 (0.43–0.94)**	**0.024**	0.141	117/105	98/140	**0.63 (0.43–0.91)**	**0.014**	**0.56 (0.37–0.83)**	**0.004**	0.039
Males	437/407	465/444	0.98 (0.81–1.18)	0.795	0.96 (0.79–1.17)	0.714		436/405	466/446	0.97 (0.81–1.17)	0.755	0.96 (0.79–1.17)	0.669	
Smoking status														
Never	224/246	209/256	0.90 (0.69–1.16)	0.406	0.87 (0.66–1.13)	0.294	0.885	223/238	210/264	0.85 (0.66–1.10)	0.212	0.83 (0.64–1.08)	0.165	0.706
Ever	331/275	353/319	0.92 (0.74–1.15)	0.455	0.90 (0.71–1.13)	0.368		330/272	354/322	0.91 (0.73–1.13)	0.381	0.88 (0.70–1.11)	0.289	
Drinking status														
Never	316/333	298/392	**0.80 (0.65–0.94)**	**0.044**	**0.79 (0.64–0.99)**	**0.042**	0.031	316/326	306/410	**0.77 (0.62–0.95)**	**0.017**	**0.75 (0.60–0.94)**	**0.012**	0.027
Ever	234/183	261/174	1.17 (0.89–1.54)	0.251	1.14 (0.85–1.51)	0.389		237/184	258/176	1.14 (0.87–1.49)	0351	1.10 (0.82–1.46)	0.532	
BMI														
<25.0	345/229	369/258	0.95 (0.75–1.20)	0.659	0.94 (0.75–1.20)	0.617	0.511	343/221	371/226	0.90 (0.71–1.13)	0.365	0.90 (0.71–1.14)	0.374	0.169
≥25.0	210/292	193/317	0.85 (0.66–1.09)	0.195	0.86 (0.66–1.10)	0.223		210/289	193/320	0.83 (0.65–1.07)	0.147	0.84 (0.65–1.08)	0.179	

Variables	rs2494752							rs10138277						
	(cases/controls)		Crude OR (95% CI)	*P*	Adjusted OR* (95% CI)	*P* a	*P*_*hom*_	(cases/controls)		Crude OR (95% CI)	*P*	Adjusted OR^a^ (95% CI)	*P* a	*P*_*hom*_
	AA	AG/GG						CC	CT/TT					
Age														
≤60	304/298	253/245	1.01 (0.80–1.28)	0.920	0.99 (0.77–1.27)	0.908	0.803	443/423	114/120	0.91 (0.68–1.21)	0.508	0.86 (0.64–1.17)	0.339	0.437
>60	307/299	253/254	0.97 (0.77–1.23)	0.801	0.97 (0.76–1.24)	0.814		455/455	105/98	1.07 (0.80–1.45)	0.657	1.07 (0.78–1.50)	0.684	
Sex														
Females	127/131	88/114	0.80 (0.55–1.15)	0.228	0.70 (0.47–1.03)	0.073	0.199	178/198	37/47	0.88 (0.54–1.41)	0.585	0.77 (0.46–1.2)	0.310	0.610
Males	484/466	418/385	1.05 (0.87–1.26)	0.644	1.04 (0.85–1.27)	0.700		720/680	182/171	1.01 (0.80–1.27)	0.965	0.98 (0.77–1.25)	0.881	
Smoking status														
Never	245/276	188/226	0.94 (0.72–1.24)	0.623	0.93 (0.72–1.22)	0.617	0.645	351/400	82/102	0.92 (0.66–1.27)	0.597	0.86 (0.62–1.21)	0.392	0.583
Ever	366/321	318/273	1.02 (0.82–1.27)	0.849	0.98 (0.78–1.24)	0.880		547/478	137/116	1.03 (0.78–1.36)	0.823	1.00 (0.75–1.34)	0.999	
Drinking status														
Never	347/385	275/351	0.87 (0.70–1.08)	0.200	0.85 (0.68–1.06)	0.147	0.039	505/582	117/154	0.88 (0.67–1.15)	0.332	0.84 (0.64–1.11)	0.218	0.159
Ever	264/212	231/148	1.25 (0.95–1.65)	0.107	1.22 (0.92–1.63)	0.174		393/296	102/64	1.20 (0.85–1.70)	0.302	1.13 (0.79–1.62)	0.508	
BMI														
<25.0	382/266	332/221	1.05 (0.83–1.32)	0.703	1.04 (0.82–1.32)	0.730	0.407	568/390	146/97	1.03 (0.78–1.38)	0.823	1.02 (0.77–1.38)	0.858	0.504
≥25.0	229/331	174/278	0.91 (0.70–1.17)	0.439	0.92 (0.71–1.18)	0.498		330/488	73/121	0.89 (0.65–1.23)	0.488	0.91 (0.66–1.26)	0.554	

a Obtained in logistic regression models with adjustment for age, sex, BMI, smoking status and drinking status.

*P*_hom_ derived from the homogeneity test.

The results were in bold, if the 95% CI excluded 1 or *P* < 0.05.

CI, confidence interval; OR, odds ratio.

Furthermore, the combined effects of these three *AKT1*SNPs were explored with data stratified by age, sex, smoking status, drinking status and BMI. We found that combined *AKT1*SNPs were significantly associated with decreased ESCC risk for women (adjusted OR = 0.56, 95% CI = 0.37–0.83, *P* = 0.004, statistical power = 0.792) and non‐drinkers (adjusted OR = 0.75, 95% CI = 0.60–0.94, *P* = 0.012, statistical power = 0.979) who carried at least one risk genotype (Table 3A). Moreover, the protective effect of combined *AKT1* SNPs (adjusted OR = 0.56, 95% CI = 0.37–0.83, *P* = 0.004) was stronger in women than that of each of *AKT1* SNP (rs2294750: adjusted OR = 0.63, 95% CI = 0.43–0.94, *P* = 0.024; rs2294752: OR = 0.70, 95% CI = 0.47–1.03, *P* = 0.073; OR = 0.77, 95% CI = 0.46–1.2, *P* = 0.310). The collectively protective effects of *AKT1* SNPs were also observed among non‐drinkers (Table 3A and B). Interestingly, logistic regression analysis discovered significantly gene–gene interactions among three *AKT1* SNPs (*P* < 0.015). These results suggested that AKT1*SNPs* might collectively protect individuals against ESCC.

### *AKT1* haplotypes and ESCC risk

We further investigated whether the haplotypes of three *AKT1* SNPs were associated with ESCC risk. As shown in Table 4, four *AKT1* haplotypes were determined in the study population. We defined the haplotype consisting of wild‐type alleles (G‐A‐C) as the reference group. The protective association was found between haplotypes C‐A‐C and ESCC susceptibility (adjusted OR = 0.70, 95% CI = 0.52–0.94). However, the results need to be further validated.

**Table 4:** Haplotype analysis for genotypes of *AKT1* and ESCC

Haplotypes^a^	Haplotype frequencies				Crude OR (95% CI)	*P*	Adjusted OR (95% CI)	*P* b
	Cases		Controls					
	*n*	%	*n*	%				
G‐A‐C	1558	69.68	1485	67.75	1.00		1.00	
C‐A‐C	87	3.89	117	5.34	**0.71 (0.53–0.94)**	**0.02**	**0.70 (0.52–0.94)**	**0.019**
C‐G‐C	362	16.19	352	16.01	0.98 (0.84–1.16)	0.837	0.97 (0.82–1.14)	0.680
C‐G‐T	227	10.15	216	9.85	1.00 (0.82–1.22)	0.987	0.97 (0.79–1.19)	0.769

a Obtained in logistic regression models with adjustment for age, sex, smoking status, drinking status and BMI.

b The results were in bold, if the 95% CI excluded 1 or P < 0.05.

### High‐order interactions in ESCC risk by MDR analysis

The MDR analysis was carried out to further explore the high‐order interactions of SNPs and environmental factors in ESCC risk. Five studied SNPs and five risk factors (*i.e*. age, sex, smoking status, drinking status and BMI) entered the analysis. BMI was shown to be the best one‐factor model, as it had the highest cross‐validation consistency (CVC, 100%) and the lowest prediction error (39.4%) out of all 10 factors. It indicated that among all factors, BMI conferred the highest ESCC risk in the study population. Moreover, when compared to other models (*e.g*. five‐factor mode and seven‐factor model), the 10‐factor model, having a maximum CVC (100%) and a minimum prediction error (33.7.0%), could yield a better prediction for ESCC risk (Table 5).

**Table 5:** MDR analysis for the risk of ESCC prediction with and without *AKT1&AKT2* variant genotypes

Best interaction models	Cross‐validation	Average prediction error	*P* a
1	100/100	0.396	<0.0001
1, 2	100/100	0.396	<0.0001
1, 2, 3	100/100	0.386	<0.0001
1, 2, 3, 4	97/100	0.380	<0.0001
1, 2, 3, 4, 5	100/100	0.370	<0.0001
1, 2, 3, 4, 5, 6	99/100	0.364	<0.0001
1, 2, 3, 4, 5, 6, 7	97/100	0.355	<0.0001
1, 2, 3, 4, 5, 6, 7, 8	100/100	0.344	<0.0001
1, 2, 3, 4, 5, 6, 7, 8, 9	100/100	0.340	<0.0001
**1, 2, 3, 4, 5, 6, 7, 8, 9, 10**	100/100	0.337	<0.0001

a *P*‐value for 1000‐fold permutation test.

The best model with maximum cross‐validation consistency and minimum prediction error rate was in bold.

Labels: 1, BMI; 2, gender; 3, smoking status; 4, age; 5, drink status; 6, rs2304186; 7, rs2494752; 8, rs2494750; 9, rs10138277; 10, rs7254617.

MDR, multifactor dimensionality reduction.

### Correlation between *AKT1* rs2494750 genotypes and *AKT1* mRNA expression levels

**Figure 1:**
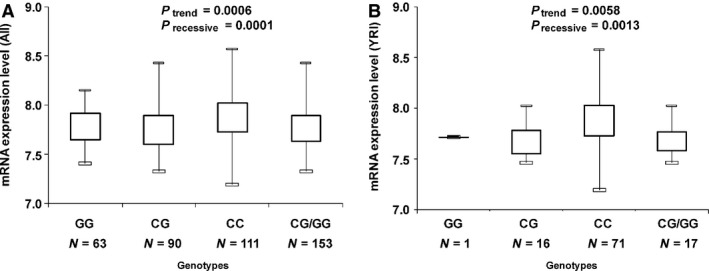
The relative expression levels of the *AKT1* gene by the *AKT1* rs2494750 genotypes in 270 HapMap participants. (**A**) *AKT1* gene expression levels under the additive model (one‐way anova analysis *P* = 0.0006) and recessive model (Student's *t*‐test, *P* = 0.0001) among the general population. (**B**) *AKT1* gene expression levels under the additive model (one‐way anova analysis *P* = 0.0058) and recessive model among YRI population (Student's *t*‐test, *P* = 0.0013).

Finally, 264 of 270 individuals were informative for analysis, of whom there were 63, 90 and 111 carriers of GG, CG and CC genotypes respectively. We found that *AKT1* rs2949750 variant C allele was significantly associated with increased *AKT1* gene expression levels under the additive model (one‐way anova, *P* = 0.0006) and recessive model (Student's *t*‐test, *P* = 0.0001; Fig. 1A). Further analysis by population group indicated that significant impact of the variant on gene expression was only observed among YRI (Fig. 1B; one‐way anova, *P* = 0.0058; Student's *t*‐test, *P* = 0.0013), rather than CEU, CHB and JPT populations (data not shown).

### Meta‐analysis for the association of *AKT1* rs2494750 and *AKT2* rs7254617 with cancer risk

**Figure 2:**
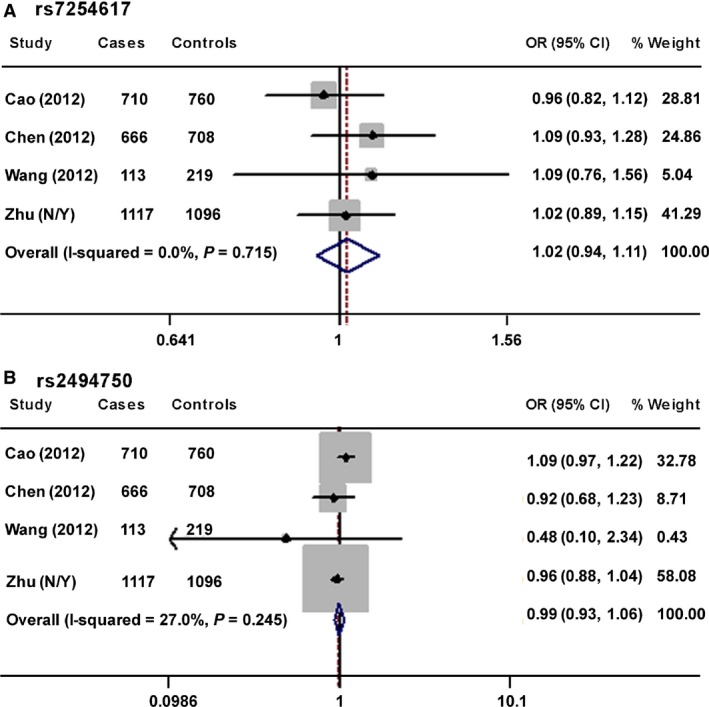
Forest plots for the mini meta‐analysis. It evaluated the associations of ESCC cancer risk with *AKT2* rs7254617 (**A**) and *AKT1* rs2494750 (**B**) under the dominant model. The size of the grey box was proportional to the percentage of weight of each study.

Thus far, three publications have reported conflicting results on the associations of *AKT1* rs2494750 and *AKT2* rs7254617 with cancer risk 9, 14, 19. With the inclusion of all these studies and our data, we carried out a mini meta‐analysis composed of 2606 cases and 2783 controls. Pooled analysis provided no evidence of the association of these two SNPs and cancer susceptibility (rs2494750 under dominant model: OR = 0.99, 95% CI = 0.93–1.06; rs7254616 under the dominant model: OR = 1.02, 95% CI = 0.94–1.11) (Fig. 2). No publication bias was detected for *AKT2* rs7254617, but significant publication bias was detected for rs2494750.

## Discussion

Oesophageal squamous cell carcinoma, with a 5‐year survival rate of less than 20% 1, 3, is the fourth leading cause of cancer‐related death in China 34. Excessive activity of the PI3K–AKT pathway is involved in carcinogenesis. AKT acts as a serine/threonine kinase downstream of PI3Ks. It is frequently constitutively activated in a wide spectrum of human cancers, including ESCC 18. Previous studies have reported that SNPS in *PI3K* and *mTOR* genes within the AKT pathway modulate the risk of various cancers 8, 9, 10, 11, 19, 20, 21, 22. SNPs that influence the activity of AKT may also modify the risk of ESCC. Therefore, we searched potentially functional SNPs in the *AKT* genes and studied for their association with ESCC susceptibility. The single‐locus analysis did not provide evidence of statistically significant association between ESCC risk and the five studied SNPs. Moreover, our meta‐analysis observed no association of *AKT1* rs2494750 and *AKT2* rs7254617 and ESCC risk. However, the combined analysis of three *AKT1*SNPs identified that individuals carrying only one of three *AKT1* variant genotypes might have decreased risk to develop ESCC cancer in comparison with non‐carriers, but this finding could be because of chance. It was noted that significant publication bias was detected in the mini meta‐analysis for rs2494750. One reason for the publication bias was that medical findings with statistical significance have greater chance to be polished than those not significant. The limited number of eligible studies might be another reason for publication bias. The resulting bias could cause erroneous conclusions 39. Thus, our meta‐analysis results call for further validation.

The effects of some *AKT* SNPs on cancer risk have been investigated previously 9, 19, 20, 21, 22. A study conducted among Caucasians reported that two SNPs in the *AKT3* gene had profound effects on bladder cancer susceptibility 20. *AKT3* rs2994329 was shown to significantly increased bladder cancer risk, while *AKT3* rs12045585 exhibited reverse association 20. The same group also reported that *AKT2* 3730050 was significantly associated with the survival of muscle invasive and metastatic bladder cancer patients 40. When compared with those with the wild‐type genotype, patients carrying one or two *AKT2* 3730050 variant alleles had an increased death risk up to 2.99‐fold 40. Recently, one study demonstrated that *AKT1* rs1130214 and rs3803300 were associated with oral squamous cell carcinoma in Chinese Han Population 21. Zhang *et al*. genotyped five *AKT1* SNPs (rs3803300, rs1130214, rs3730358, rs1130233 and rs2494732) in 593 nasopharyngeal carcinoma cases and 480 controls 22. Although none of individual SNP had significant effect on the risk of nasopharyngeal carcinoma, a two‐SNP haplotype, consisting variant alleles of *AKT1* rs1130233 and rs2494732, was significantly associated with increased nasopharyngeal carcinoma risk 22. Moreover, both *AKT1* rs2294750 and *AKT2* rs7254617 polymorphisms have been investigated in cancers among Chinese populations 9, 19, but results are conflicting. Cao *et al*. reported there was no association between renal cell cancer risk and these two SNPs, but a stratification analysis was not performed 19. Chen *et al*. reported that *AKT2* rs7254617, but not *AKT1* rs2294750, significantly increased the risk of prostate cancer 9. Taken together, the majority of studies 19, 20, 21, 22 support *AKTs* as cancer susceptibility genes. The inconsistency among results may be because of the discrepancies in the sampling, different ethnicity or the fact that polymorphisms in *AKT* genes may play a tissue‐specific role in the carcinogenesis.

*AKT1* rs2294750 SNP show a significantly reverse association with ESCC risk among non‐drinkers, but not among drinkers. Alcohol, considered as class I carcinogen by International Agency for Research 41, is one of most remarkable risk factor for ESCC carcinogenesis. The underlying mechanism of how alcohol affects the development of ESCC remains unclear. It may directly irritate the epithelium of oesophagus, enhance vulnerability to another carcinogen or cause nutrition deficiencies that are also a recognized risk factor for ESCC 42. Despite lack of the mechanism, epidemiologic evidence has consistently shown that alcohol use is associated with an increased ESCC risk 41, 42, 43, 44, 45. As an example, alcohol consumption exceeding the recommended U.S. dietary guidelines is significantly associated with elevated ESCC risk 41. The protective effects of *AKT1* rs2294750 on non‐drinker observed in this study is in accordance with the perception of cancer susceptibility, which represents a genetic attribute that modify the possible cancer risk under the influence of environmental conditions or lifestyles, such as smoking, drinking and diet. Given the aetiological role of drinking in the development of ESCC, the moderate protective effect of *AKT1* rs2294750 on drinker is probably overridden by the potent carcinogenic effect of alcohol. Alternatively, among non‐drinkers without alcohol's damaging effects, the SNP was able to significantly decrease ESCC risk.

Moreover, we found that *AKT1* rs2294750 had a protective effect on women against ESCC risk. Previous epidemiology studies demonstrated the conspicuous male preponderance of ESCC 1, 2, 46, which suggests that males appear to be predisposed to environmentally induced ESCC, compared with female. Comparable to the results observed in stratified analysis by drinking status, the protective impact of *AKT1* rs2294750 was also more predominant in low‐risk subgroup (women) than in high‐risk subgroup (males). These data may suggest that the protective effect of this SNP on men might be superseded by unknown sex‐related environmental aetiology, which could be resulted from gene–environment interaction 47 that needs to be detected in a large study. In the current studies, significant associations were only observed in women and non‐drinkers, indicating the importance in considering other factors when investigating genotypic impact on cancer susceptibility. Alternatively, these results could be because of chance, which call for larger and validation studies. The relative gene expression analysis by HapMap genotypes demonstrated that *AKT1* rs2949750 variant C allele was significantly associated with elevated *AKT1* gene expression levels among the general population and the YRI population but not other three subpopulations.

Finally, although there was no association between ESCC susceptibility and any of *AKT1* variants in the single‐locus analysis, our results revealed that three *AKT1* SNPs might collectively protect individuals from developing ESCC. First, among women and non‐drinkers, the observed combined protective effect of the three *AKT1* SNPs was stronger than each of individual SNPs. Second, significant gene–gene interaction among three *AKT1* SNPs was identified by logistic regression analysis. Third, a three‐*AKT1* SNP haplotype was significantly associated with ESCC risk. The lack of main effect of *AKT1* variants might suggest that the effect size of any of the variants under investigation was small and the current sample size was not large enough to detect such small effects. It might also suggest that these SNPs were low penetrance variants that modulate cancer susceptibility through gene–gene and/or environment–gene interactions. Moreover, the combined analysis is able to amplify the moderate effect of each individual SNP and enhance the predictive power. The identification of multiple risk variants may therefore improve risk prediction and could conceivably be applied to assessment of an individual's ESCC risk. As indicated by the online tool SNPinfo software, *AKT1* rs10138277 and rs2494750 are SNPs in the transcription factor‐binding site of the gene and these SNPs may alter the binding capacity of the related transcription factors. *AKT1* rs2494752 was selected because that it was reported to be associated with chemotherapy response in advanced non‐small cell lung cancer among a Chinese Population 25, and it is also a SNP in the transcription factor‐binding site of the *AKT1* gene. The MDR analysis further validated the observed gene–gene and gene–environment interaction by logistic regression analysis, in which 10‐factor model consisting of SNPs and environmental factors could more accurately predict ESCC risk than any SNP or environmental factor alone. ESCC is known as a complex, multifactorial disease, in which interplay between genetic and environment factors may play a crucial role, and one single SNP is not adequate to predict the overall risk. However, the combination of susceptible loci in multiple biological pathways and environmental factor may help health profession improve predictions of the overall risk and clinical outcome, identification of high‐risk subpopulation and early detection for ESCCs.

There are some limitations in this study. First, although age, sex, smoking, drinking and BMI were considered and adjusted for in the multivariate analysis, many other factors (nutrition, diet, socio‐economic status, *etc*.) that may also modulate predisposition to ESCC were not available for the analysis; Lack of the detailed data limited our ability to explore gene–gene and gene–environment interactions. Second, ESCC patients were only recruited from Fudan University Shanghai Cancer Center, the case–control study might suffer from selection bias and information bias. Third, this study only had moderate sample size, which might compromise our ability to detect relatively weak main effect or interactions of some potentially functional SNPs. Fourth, the statistical power for the stratification analysis and determination of gene–gene and gene–environmental interaction might be limited. Moreover, our findings from observational association studies may require *in vitro* and *in vivo* experiments to further provide biological evidence of the observed protective effects of AKT1 SNPs on ESCC risk, which would unravel the underlying molecular mechanisms. As a result, our results should be carefully interpreted.

In summary, we found that *AKT1* rs2294750 alone or together with other two *AKI* SNPs may modify the susceptibility to ESCC risk; nevertheless, these effects were largely dependent on the presence of other risk factors, *i.e*. sex and drinking status. Our results draw attention to the importance of gene–gene and gene–environment interactions in determining the ESCC susceptibility. These genetic variants may cause an individual susceptible to certain effects of environmental factors. Larger population‐based studies, with a focus on gene–environment interaction, are needed to substantiate our findings.

## Conflicts of interest

The authors confirm that there are no conflicts of interest.
